# End‐expiratory lung volume remains stable during N_2_ MBW in healthy sleeping infants

**DOI:** 10.14814/phy2.14477

**Published:** 2020-08-26

**Authors:** Per M. Gustafsson, Laszlo Kadar, Sanna Kjellberg, Lena Andersson, Anders Lindblad, Paul D. Robinson

**Affiliations:** ^1^ Department of Pediatrics Central Hospital Skövde Sweden; ^2^ The Sahlgrenska Academy at the University of Gothenburg Gothenburg Sweden; ^3^ CF Centre Queen Silvia Children's Hospital Gothenburg Sweden; ^4^ Department of Respiratory Medicine The Children’s Hospital at Westmead Sydney NSW Australia; ^5^ Discipline of Paediatrics and Child Health Sydney Medical School University of Sydney Sydney NSW Australia

**Keywords:** breathing pattern, functional residual capacity, inert gas washout, respiratory inductance plethysmography, ultrasonic flow sensor

## Abstract

We have previously shown that functional residual capacity (FRC) and lung clearance index were significantly greater in sleeping healthy infants when measured by N_2_ (nitrogen) washout using 100% O_2_ (oxygen) versus 4% SF_6_ (sulfur hexafluoride) washout using air. Following 100% O_2_ exposure, tidal volumes decreased by over 30%, while end‐expiratory lung volume (EELV, i.e., FRC) rose markedly based on ultrasonic flow meter assessments. In the present study to investigate the mechanism behind the observed changes, N_2_ MBW was performed in 10 separate healthy full‐term spontaneously sleeping infants, mean (range) 26 (18–31) weeks, with simultaneous EELV monitoring (respiratory inductance plethysmography, RIP) and oxygen uptake (V´O_2_) assessment during prephase air breathing, during N_2_ washout by exposure to 100% O_2_, and subsequently during air breathing. While flow meter signals suggested a rise in ELLV by mean (*SD*) 26 (9) ml over the washout period, RIP signals demonstrated no EELV change. V'O_2_/FRC ratio during air breathing was mean (*SD*) 0.43 (0.08)/min, approximately seven times higher than that calculated from adult data. We propose that our previously reported flow meter‐based overestimation of EELV was in fact a physiological artifact caused by rapid and marked movement of O_2_ across the alveolar capillary membrane into the blood and tissue during 100% O_2_ exposure, without concomitant transfer of N_2_ to the same degree in the opposite direction. This may be driven by the high observed O_2_ consumption and resulting cardiac output encountered in infancy. Furthermore, the low resting lung volume in infancy may make this error in lung volume determination by N_2_ washout relatively large.

## Graphical abstract

In infancy, 100% O_2_ exposure during MBW decreased tidal volumes by >30%, whilst end‐expiratory lung volume (EELV, i.e., FRC) rose markedly, based on ultrasonic flow meter assessments. Respiratory inductance plethysmography signals demonstrated no EELV change. We propose this flow meter EELV overestimation is a physiological artifact caused by rapid marked O_2_ movement across the alveolar capillary membrane into blood and tissue during 100% O_2_ exposure, without concomitant equivalent N_2_ transfer in opposite direction. High O_2_ consumption and cardiac output in infancy may drive this. Low resting lung volume in infancy may make this lung volume determination error by N_2_ MBW relatively large.

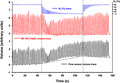

## 1 INTRODUCTION

The multiple‐breath inert gas washout (MBW) method is increasingly used in young children for early detection of lung disease and to assess the impact on lung function from serious conditions such as cystic fibrosis (CF). Its high sensitivity to detect peripheral airway impairment from infancy (Amin et al., 2010; Gustafsson, De Jong, Tiddens, & Lindblad, 2008; Lum et al., 2007) is combined with high feasibility in all age groups (Robinson et al., 2018). The lung clearance index (LCI) from the MBW test has been recently endorsed as a primary outcome measure in CF clinical trials (Kent et al., 2014). The impact of inert gas choice remains of great interest (Guglani et al., 2018; Gustafsson, Bengtsson, Lindblad, & Robinson, 2017) and nitrogen‐based (N_2_) MBW using 100% oxygen (O_2_) continues to be advocated for this infant age group (Koucky, Skalicka, & Pohunek, 2018). Our work has focused on defining the physiological impact of 100% O_2_ exposure during MBW in this age group. Our initial study, published in the *Journal of Applied Physiology* in 2017, reported results from a study in 10 healthy full‐term spontaneously sleeping infants, comparing FRC (functional residual capacity), LCI, and breathing pattern during MBW performed with two different inert gas choices, nitrogen‐based (N_2_) MBW using 100% oxygen (O_2_) and sulfur hexafluoride (SF_6_) washout by air using the same recording device (Gustafsson et al., 2017). We demonstrated that both measured FRC and indices of ventilation inhomogeneity (i.e., LCI) were significantly greater using N_2_ washout versus SF_6_ MBW. Importantly, 100% O_2_ exposure during N_2_ MBW was associated with significant changes in the breathing pattern of the infants studied. These were not observed during SF_6_ MBW where O_2_ exposure was kept constant at 21%.

Exposure to 100% O_2_ led to a transient significant reduction in tidal volume (V_T_), minute ventilation (V′_E_), and “respiratory drive” (mean inspiratory flow; V_T_in/Tin) with a concomitant increase in end‐tidal CO_2_, while respiratory rate was unchanged (Koucky et al., 2018). A significant rise of the end‐expiratory lung volume (EELV, i.e., FRC) by a mean of 19 ml (≈10% relative) occurred based on the cumulative difference between inspiratory and expiratory tidal volumes (V_T_in ‐ V_T_ex) measured by the ultrasonic flow meter over the course of the N_2_ washout phase. The lack of change during in vitro N_2_ MBW testing was consistent with an apparent physiological effect. Previous explanations for the fall in V_T_ and V’_E_ include “unloading” of the peripheral chemoreceptors leading to reduced “respiratory drive”, however, this was the first clear description in the literature of a change in EELV with 100% O_2_ exposure. Another potential explanation for this marked physiological artifact is the excess movement of O_2_ across the alveolar capillary membrane driven by the high metabolic rate or O_2_ consumption present in infants, due to a high O_2_ consumption per unit of alveolar gas volume.

Respiratory inductance plethysmography (RIP) is a method for monitoring breathing pattern based on the use of two elastic belts containing insulated sinusoid wire coils (Konno & Mead, 1967). These transducer bands are placed around the rib cage (RC) and the abdomen (ABD) and are connected to an oscillator. The recorded frequency in the respective circuits is proportional to the cross‐sectional area of the body at their respective positions and changes relative to V_T_ excursions. The frequency signal is demodulated electronically to derive digital waveforms replicating the V_T_. The RIP signals can be calibrated using various maneuvers (Stromberg, Dahlback, & Gustafsson, 1993; Stromberg & Gustafsson, 2000) or they can be directly used and all changes are then relative. RIP can be run in either alternate current (AC) or direct current DC mode. In AC mode, signals have a relatively long time constant allowing the signals to return to a starting set point. This avoids volume drift and ensures good quality of V_T_ assessments even when body position is changed, but does not allow accurate monitoring of EELV changes. In DC mode, signal output is proportional to cross‐sectional body surface area with no built‐in signal time constant, making it ideal for monitoring of breathing pattern and EELV in supine sleeping subjects who do not change body posture, such as sleeping infants monitored over the course of several minutes.

The purpose of the present study was to test the hypothesis that 100% O_2_ exposure during N_2_ MBW in healthy sleeping infants does not lead to an increase in EELV as assessed using RIP. Resting oxygen consumption was derived from MBW data collected prior to the start of washout with 100% O_2_ and compared to previously collected adult data.

## 2 METHODS

### 2.1 Test subjects and ethics

We recruited a separate cohort of healthy normally developed full‐term infants without a history of congenital lung malformations or health problems other than common transient upper airway or gastrointestinal infections. Invitation letters were sent out to care‐givers of infants in East Skaraborg County, West Sweden, using addresses provided from the Swedish population register. The infants were tested in the supine posture during quiet spontaneous sleep after a feed. A prephase of stable air breathing for 30–50 s (termed “pre‐phase”) was followed by N_2_ washout using 100% O_2_ (termed “exposure phase”), and subsequent N_2_ washin during medical air breathing (termed “recovery phase”). The study was approved by the Ethics Committee of the University of Gothenburg (DNR 746‐15).

### 2.2 Recording systems and procedures

MBW testing was performed using the Exhalyzer® D (ECO Medics AG, Switzerland), which measures N_2_ indirectly from the O_2_ and CO_2_ signals recorded. The calibration and N_2_ MBW recording procedures were performed in identical manner as reported in our previous study (Gustafsson et al., 2017), to which we refer for details. Data were recorded at 200 Hz per channel. Spiroware software version 3.2.1 was used for the recordings and data analysis. An infant‐size NOX RIP thorax belt was placed around the chest at the level of the nipples (No 7072197, RC‐band) and a NOX RIP abdomen belt was placed around the abdomen at the level of the umbilicus (No 7072196, ABD‐band; NOX RIP BELTs Disposable Pediatric N0 7072086, NOX Medical Katrinartun 2, 105 Reykjavik, Iceland). A custom‐built RIP oscillator and demodulator was used, and data were recorded at 200 Hz per channel using a custom‐written LabView (National Instruments, Cambridge, UK) application. The DC‐mode RC and ABD voltage signals were added to obtain a surrogate uncalibrated V_T_ voltage signal allowing for monitoring of V_T_ excursions and EELV. The sum RIP signal was scaled and synchronized with the corresponding flow meter signals. From the recordings made in each infant one test was selected for inclusion in the final analysis based on having the best quality of N_2_ washout and RIP traces, focusing on stability of EELV and V_T_ in the prephase and lack of evidence of leaks or sighs over the washout and subsequent N_2_ washin. From a minimum of 20 breaths obtained during prephase air breathing recordings, V´O_2_, CO_2_ excretion (V´CO_2_), and RQ (Respiratory quotient, respiratory exchange ratio) were calculated after conversion of gas volumes into ambient temperature pressure dry conditions. Indirect calorimetry metabolic rate was calculated using the Weir equation (Weir, 1949): resting energy expenditure (REE) = [(3.941 × V´O_2_ (L)) + (1.106 × V´CO_2_ (L))] × 1,440 (kcal/day).

### 2.3 Data analysis and statistics

Microsoft Excel 2010 was used to compile and graphically display data. Tidal volume traces obtained from the synchronized, calibrated, and BTPS‐corrected output files (“B‐files”) using the Spiroware v3.2.1 software were visually aligned with the RIP V_T_ traces as described above. The maximum cumulative differences between inspiratory and expiratory tidal volumes (V_T_in−V_T_ex) starting with the first washout breath were calculated using Excel based on the tabulated output data from Spiroware.

## 3 RESULTS

**FIGURE 1:**
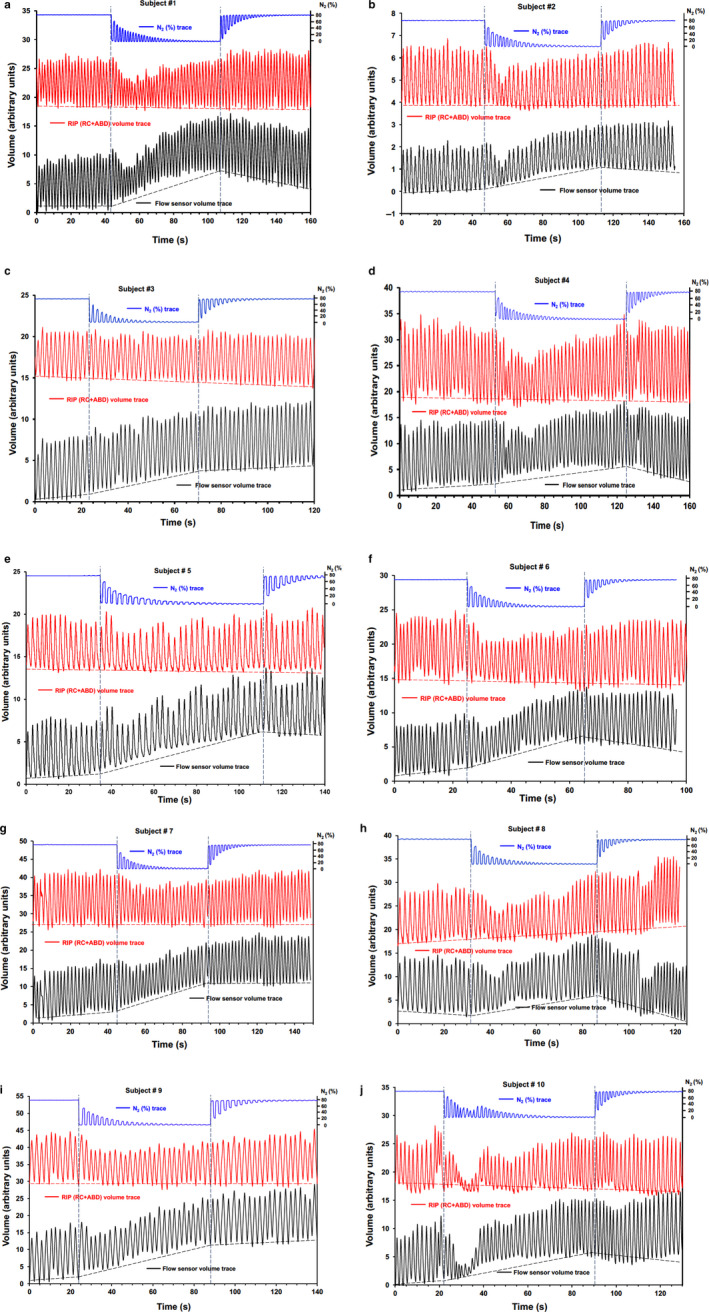
(panel a–j). N_2_ concentrations, RIP (respiratory inductance plethysmography) and flow sensor derived volume traces during air breathing prephase (stable N_2_ concentrations), 100% O_2_ exposure phase (falling N_2_ end‐tidal concentrations) in 10 infants, and subsequent air breathing recovery phase (rising N_2_ end‐tidal concentrations). Vertical dashed lines delineate the start and end of the exposure phase, while the direction of change in EELV before, during, and after the exposure phase for the flow meter and RIP signals is also shown

Twelve infants were recruited and subsequently attended the Department of Pediatrics at the Central Hospital in Skövde. Of these, 10 contributed at least one technically acceptable N_2_ MBW with simultaneous RIP recording. The remaining two subjects did not fall asleep or woke at the start of test procedures. The study group consisted of 10 healthy infants (six boys) aged mean (*SD*; range) 25 (4; 18–31) weeks) (Table 1), performing 2–4 N_2_ washout tests in the same session. Table 2 summarizes FRC, LCI, and breathing pattern results. Mean (*SD*) FRC was 129 (26) ml or 16.3 (2.7) ml/kg and LCI was 8.43 (0.75) across the cohort. During the exposure phase, V_T_ dropped initially but to varying degrees among the subjects. For the study group as a whole, minimum expiratory V_T_ during the exposure phase fell to a mean (*SD*) 47 (14)% of the maximum prephase V_T_ value, or 63 (20)%, if expressed as a proportion of median exposure phase V_T_. Figure 1 (panels a–j) summarizes the N_2_ concentration, uncalibrated combined rib cage and abdomen volume, and ultrasonic flow meter volume traces during the prephase, exposure, and recovery phases separately in all 10 participants.

**TABLE 1:** Demographic data of study population

Subject	Initials	Gender	Age (weeks)	Body weight (kg)	Height (cm)
1	AVH	Male	18	7.4	64
2	AN	Female	30	7.3	67
3	OSW	Male	24	7.4	66
4	VK	Female	23	8.6	68
5	KF	Male	23	9.1	71
6	AL	Female	21	6.5	61
7	GS	Male	28	8.9	68
8	IL	Male	29	7.4	65
9	LL	Female	31	7.8	69
10	ON	Male	25	8.6	68
Mean			25	7.9	67
*SD*			4	0.8	3

**TABLE 2:** N_2_ multiple‐breath washout results

Subject	FRC (ml)	LCI	Number of washout breaths	V_T_ex (ml)			Cumulative V_T_in−V_T_ex difference (ml)	Number of tests
				Median	Max	Min		
1	117	7.85	32	35	48	21	31	2
2	120	7.68	23	49	58	24	20	2
3	155	9.55	25	64	99	55	20	2
4	144	8.78	32	45	59	26	12	3
5	165	7.50	24	58	78	46	27	3
6	81	8.24	23	35	50	26	24	2
7	124	8.55	24	49	59	33	30	4
8	96	9.60	24	47	64	30	22	3
9	146	7.76	22	55	70	40	43	4
10	138	8.75	33	47	63	7	32	4
Mean	129	8.43	26	48	65	31	26	3
*SD*	26	0.75	4	9	15	14	9	1

Abbreviations: FRC, functional residual capacity; LCI, lung clearance index; V_T_ex, expired tidal volume; V_T_in, inspired tidal volume.

A very close agreement between the RIP and flow meter based V_T_ excursions was visually confirmed. Overall, the prephase showed a stable EELV as recorded by the two independent methods. In contrast, EELV appeared to rise over the first half or two thirds of the washout as recorded by the flow meter, followed by a stable or falling EELV. However, RIP signals remained consistent with a stable EELV. The maximum cumulative difference in flow meter based inspiratory and expiratory V_T_ was mean (*SD*; range) 26 (9; 12–43) ml (Table 2), constituting a mean (*SD*; range) 52 (20)% of median expiratory V_T_ and 20 (7)% of measured FRC (mean (*SD*)), for the study group as a whole.

Mean (*SD*) V´O_2_ and V´CO_2_ during air breathing were 6.75 (0.67) and 6.12 (0.99) ml min^−1^ kg^−1^ body weight, respectively, giving an RQ of mean (*SD*) 0.91 (0.13) (Table 3). The REE (metabolic rate) was mean (*SD*) 48.0 (4.7) kcal 24 h^−1^ kg^−1^ body weight.

**TABLE 3:** Metabolic results from indirect calorimetry during prephase air breathing

Subject number	V'O_2_		V'CO_2_		RQ	Metabolic rate	
	(ml/min)	(ml min^−1^ kg^−1^)	(ml/min)	(ml min^−1^ kg^−1^)		(kcal/24 h)	(kcal 24 h^−1^ kg^−1^)
1	49.06	6.65	44.35	6.01	0.90	349	47.3
2	47.00	6.44	43.15	5.91	0.92	336	46.0
3	54.25	7.36	58.46	7.93	1.08	401	54.4
4	66.48	7.15	43.71	4.70	0.66	447	48.1
5	47.27	5.22	43.44	4.79	0.92	337	37.2
6	45.66	7.02	39.84	6.13	0.87	323	49.6
7	55.18	6.19	62.84	7.05	1.14	413	46.4
8	52.66	7.12	43.54	5.88	0.83	368	49.8
9	57.97	7.47	54.35	7.00	0.94	416	53.5
10	59.02	6.83	50.42	5.84	0.85	415	48.1
Mean	53.45	6.75	48.41	6.12	0.91	380	48.0
*SD*	6.54	0.67	7.72	0.99	0.13	43	4.7

Abbreviations: RQ, respiratory quotient or respiratory exchange ratio; V´CO_2_, CO_2_ excretion; V´O_2,_ oxygen uptake. Data presented as measured and per kg body weight values.

## 4 DISCUSSION

### 4.1 Summary of findings

The present study confirms our previously published observations (Gustafsson et al., 2017) of a marked reduction in V_T_ and an apparent rise in EELV over the course of an N_2_ MBW using 100% O_2_ for washout of N_2_ in a separate cohort of healthy infants. By using concomitant flow meter and RIP recordings in the present study, we have confirmed our hypothesis that the rise in EELV during washout, based on the flow meter signal, represents a physiological artifact and not a true change in EELV. This has not been clearly described before in the literature. Our findings provide further evidence to support the view that N_2_ MBW using 100% O_2_ is not the MBW method of choice in infancy. The hypothesis that significant transfer of gas species across the alveolar capillary membranes plays a significant factor in the larger N_2_ versus SF_6_ FRC and LCI values in infants previously reported (Gustafsson et al., 2017) is further supported by the greater REE (metabolic rate) values observed in these infants studied, compared to previously published values for adults.

### 4.2 Interpretation of findings

In our previously reported study comparing N_2_ and SF_6_ MBW results in healthy spontaneously sleeping infants (Gustafsson et al., 2017) in vitro tests demonstrated that inspiratory and expiratory V_T_ difference during 100% O_2_ exposure was not due to a technical artifact. We propose that a large influx of O_2_ into the blood and further into other body tissue, not balanced by a corresponding N_2_ outflow, might explain the apparent changes in EELV. While partial pressure of N_2_ in blood or other tissue is similar to that in the atmosphere, O_2_ partial pressure is lower in blood and tissue than in the atmosphere. The influx of O_2_ is driven by the high resting metabolic rate, measured in our infants, and as a result they have a high cardiac output for body and lung size (FRC).

Our estimates of resting metabolic rate are consistent with the previously published literature in the age range, with reported values within 15% of those obtained using published prediction equations (Brody, 1945; Lindahl, 1989). Lindahl studied 38 children aged 1 day to 7 years during anesthesia and reported prediction equations based on the fact that metabolism was related to body weight: for the overall cohort, V´O_2_ = 5.0 × kg + 19.8 (*r* .94) and V´CO_2_ = 4.8 × kg + 6.4 (*r* .94); in those <10 kg (*n* = 21), V´O_2_ = 6.8 × kg + 8.0 (*r* .78) and V´CO_2_ = 7.2 × kg + −8.9 (*r* .90); RQ was 0.7–1.0 (Lindahl, 1989). Compared to predicted values, our measured values underestimated V´O_2_ by 9.9% and 13.4% (<10 kg specific equation) and overestimated V´CO_2_ by 9.2% and 0.4%, respectively. Compared to predicted values based on the Brody equation (V´O_2_ = 10*BW^3/4^, where BW is body weight in kg) V´O_2_ was overestimated by 13.5%. This latter Brody equation is, however, based on lean body weight. In our studies, the accuracy of O_2_ and CO_2_ flow measurements is further supported by the fact that both are used in assessments of FRC N_2_ and FRC SF_6_ using the Exhalyzer® D device, and FRC measurement validated accuracy has been demonstrated in vitro previously (Gustafsson, Robinson, Lindblad, & Oberli, 2016; Singer, Houltz, Latzin, Robinson, & Gustafsson, 2012).

In the present study the mean (*SD*) ratio of V´O_2_ to FRC in the infants was 0.43 (0.08)/min. Subtraction of airway dead space (estimated as 2 ml/kg body weight) to calculate V_A_ (alveolar volume) elevated this ratio to 0.49 (0.11). This is greater than previous estimates for this age range. Dharmakumara, Prisk, Royce, Tawhai, and Thompson (2014) reported values of approximately 1.11/min for V´O_2_/V_A_ in mice, as representative for small animals. The authors estimated V´O_2_/V_A_ in an adult male to be approximately 0.04/min (Dharmakumara et al., 2014), and proposed similar V´O_2_/V_A_ values in children (≈0.10/min). Infants and neonates were thought to have values of about 0.20/min, based on the Lindahl (1989) study data.

Agreement with this trend is also illustrated from derived adult data for resting V´O_2_/FRC ratio. Cunha *et al* measured resting V´O_2_ in 125 healthy males (aged 17–38 years) and reported mean (95% confidence interval) values of 3.21 (3.13–3.30) ml kg^−1^ min^−1^ (Cunha, Midgley, Montenegro, Oliveira, & Farinatti, 2013). In our lung function testing laboratory, the N_2_‐derived MBW FRC values measured in an adult healthy control dataset (*n* = 137, aged 18–40 years, 66 females) were mean (*SD*) 48.7 (12.3) ml/kg body weight. Combining these two mean values of the datasets, and subtracting 200 ml airway dead space from FRC to derive V_A_, produces an adult V´O_2_/V_A_ estimate of 0.066/min. These comparative data suggest that V´O_2_/V_A_ was approximately 7.4 times greater in the infants studied than this adult estimate (0.49 vs. 0.066), supporting our hypothesis that a relatively large O_2_ influx in relation to both resting lung volume and N_2_ efflux could explain EELV drift and overestimation using the flow sensor. We have not directly compared the flux of O_2_ and N_2_ as N_2_ is indirectly measured using the combined O_2_ and CO_2_ signals with Exhalyzer® D. We believe, however, that the relatively greater influx of O_2_ into tissue of the infants is to some degree balanced by tissue N_2_ being transported into the lungs and further exhaled. This may account for the greater FRC and LCI using N_2_ versus SF_6_ MBW infants previously reported (Gustafsson et al., 2017).

### 4.3 Strengths and weakness with the present study

The present study was undertaken in sleeping healthy infants during spontaneous sleep. By avoiding the influence of sedation or lung disease, these findings are very likely to reflect true physiological events provoked by exposure to 100% O_2_. The narrow age range of the infants studied (18–31 weeks) reflects attempts made to obtain successful sleep induction after a daytime meal, but did not allow us to investigate whether there is an age influence on the “apparent EELV effect” reported. It will be of interest to explore this in future studies and to explore if there is a correlation between the magnitude of this EELV difference and the difference between N_2_ versus SF_6_ FRC and LCI indices in a given subject. Due to limited infant sleep time of the infants studied, sufficient high‐quality data to allow formal comparison of within‐subject variability of RIP data were not obtained, nor was concurrent SF_6_ MBW testing with RIP attempted. However, in our earlier study no EELV effect was observed with flow meter signals during SF_6_ MBW, and is therefore unlikely to be present on RIP signals.

RIP signals were not directly calibrated against a flow meter (Konno & Mead, 1967; Stromberg et al., 1993; Stromberg, Eklund, & Gustafsson, 1996) due to its time consuming nature and potential detrimental impact and the ability of the child to maintain quiet sleep for sufficient MBW testing. A previous study has validated the use of uncalibrated RIP signals to determine infant tidal breathing indices (Stick, Ellis, LeSouef, & Sly, 1992), and the close agreement between flow meter and RIP V_T_ traces reinforce our approach that calibration of RIP was not necessary for obtaining trustworthy replications of the true EELV course during washout. Two infants (subject # 6 and 8; Table 2) had markedly lower FRC than the remainders. The parents of these infants did not report any history of respiratory problems and none of the participants was born prematurely. Possible explanations include overfeeding before testing and/or variation in sleep state such that EELV was not actively maintained, in contrast to the normal dynamic lung volume control exerted by young infants (Kosch & Stark, 1984). Nevertheless, the pattern of V_T_ and “apparent EELV changes” were similar in these two infants compared to the remaining participants.

Formal studies comparing EELV and V_T_ course over N_2_ MBWs with 100% O_2_ in older subjects during sleep or wakefulness and in different body postures are lacking. Firm conclusions about the generalizability of present findings in spontaneously sleeping supine infants to older age groups can therefore not be drawn and further studies are warranted. Ideally, direct measurements of N_2_ and O_2_ in and out flux should be made. Comparisons of cardiac output and metabolic rate across childhood during or in very close time with N_2_ and SF_6_ MBW would be of great interest to test the idea that a high cardiac output in relation to body size or lung size is a driving mechanism of the tissue N_2_ causing a greater difference between N_2_‐ and SF_6_‐derived FRC and LCI values in younger ages.

### 4.4 Summary and conclusions

Based on ultrasonic flow meter signals, EELV appeared to rise markedly in sleeping healthy infants over the course of an N_2_ washout using 100% O_2_ and then stabilize or decline over subsequent N_2_ washin. Simultaneous DC‐mode RIP recordings, however, demonstrate that EELV remains stable over the washout. We propose that a rapid and marked movement of O_2_ across the alveolar capillary membrane into the blood and tissue occurs during pure O_2_ exposure, without concomitant transfer of N_2_ to the same degree in the opposite direction. The cause of this may be the relatively high V´O_2_ and cardiac output of infants in relation to their resting lung volume. Modeling studies in the future would be of great value to investigate this mechanism further.

## AUTHOR CONTRIBUTIONS

Per M. Gustafsson initiated, designed, and planned the study, developed some of the recording devices and software, and analyzed data. Laszlo Kadar, Sanna Kjellberg, Lena Andersson, Anders Lindblad, and Paul D. Robinson participated in the design and planning of the study. Laszlo Kadar, Sanna Kjellberg, and Lena Andersson recruited test subjects and recorded data, and performed preliminary reviews of recorded data. Per M. Gustafsson performed the final analysis of data and drafted the manuscript together with Paul D. Robinson. All authors contributed significantly to the final writing of the manuscript.
